# Involvement of Anion-Specific Effects in Changes in the Gelation and Thermodynamic Properties of Calcium Alginate Hydrogel

**DOI:** 10.3390/foods14040634

**Published:** 2025-02-13

**Authors:** Yuqiao Wang, Lin Li, Jiacheng Liu, Jianan Yan, Ce Wang, Bin Lai, Yu Dong, Haitao Wu

**Affiliations:** 1 SKL of Marine Food Processing & Safety Control, National Engineering Research Center of Seafood, Collaborative Innovation Center of Seafood Deep Processing, School of Food Science and Technology, Dalian Polytechnic University, No. 1, Qinggongyuan, Dalian 116034, China; wangyuqiao1231@163.com (Y.W.); lilin2520@163.com (L.L.); 13332254935@163.com (J.L.); yjn3vv@163.com (J.Y.); wangceyx@163.com (C.W.); laibin@dlpu.edu.cn (B.L.); 2 Dalian Feide Biological Industry Co., Ltd., Dalian 116085, China; 13591320855@163.com

**Keywords:** alginate hydrogel, anion-specific effect, M/G ratios, calcium salt type

## Abstract

The gelation process and hydrogel properties of calcium salt-induced alginate hydrogels are influenced by anion-specific effects. In this study, we investigated the effects of CaSO_4_, CaI_2_, and Ca(C_5_H_9_O_3_)_2_ [calcium β-hydroxy-β-methylbutyrate, CaHMB] on the gelation behavior of alginate hydrogels, using various mannuronic/guluronic acid (M/G) ratios to elucidate the underlying mechanisms of anion-specific effects. Here, at a high M/G ratio (2:1), the gelation time of CaSO_4_, as a low-solubility calcium source, delayed the formation of the calcium alginate hydrogel. The gelation time was 1.8 times that of the high-solubility calcium source CaHMB. Strongly hydrated ions (such as SO_4_^2−^ and C_5_H_9_O_3_^−^) caused the removal of water molecules from polysaccharide chains, resulting in the formation of small pores on the pore wall. Moreover, weakly hydrated chaotropic anions (I^−^) promoted the binding of alginate polysaccharide chains and water molecules, resulting in the slower thermal decomposition of water inside the gel. However, when the M/G ratio was reduced to 1:1 or 1:2, the influence of the three calcium salts on the water and thermodynamic properties of the hydrogels decreased, indicating that the anion-specific effect weakened. This study highlights the importance of anion-specific effects on the properties of alginate hydrogels and provides insights into the utilization of these effects to fabricate functional hydrogels with variable properties.

## 1. Introduction

Alginate (ALG) is a water-soluble polyanionic polymer and is used in industries, ranging from manufacturing to medicine and food production, owing to its favorable characteristics, including non-toxicity, biocompatibility, biodegradability, and ease of use [1]. The structure of ALG is typically described in terms of the mannuronic/guluronic acid (M/G) ratios, which play a crucial role in determining chemical, biological, functional, and physical characteristics. The G residues provide a substantial degree of stiffness and serve as the primary unit for stabilizing intermolecular structures and forming ionic crosslinks [2]. The M residues display a degree of flexibility, yet their high steric barrier renders ion crosslinking challenging, and the maintenance of stable crosslinked structures is also difficult [3]. Furthermore, a notable characteristic of ALG is its ionotropic gelation mechanism, which utilizes Ca^2^⁺ ions to form hydrogels [4]. Specific interactions between divalent ions and alginate-related poly-G, which also involve the periodically alternating copolymers poly-MGM and poly-M, influence thermodynamic properties [5]. Therefore, it is worth noting that the physicochemical properties of calcium alginate hydrogel are intricately intertwined with factors such as the M/G ratio, the overall molecular conformation, and the calcium salt type.

The Hofmeister effect, also known as the ion-specific effect, has applications in areas including ice nucleation, recrystallization, colloidal assembly, and surface tension [6]. This ion-specific effect is primarily driven by the precise interaction of ions with water molecules and their influence on the hydrogen-bonding network of water. Ions are categorized as either kosmotropic with strong hydration properties or chaotropic with weak hydration properties to account for ion-specific hydration [7]. Owing to their significant impact on water-soluble macromolecules, salts are also employed to assess the attributes of hydrogels [8]. This has also been detected in various natural polysaccharide solutions, such as galactomannan [9], guar gum, sodium hyaluronate [10], and starch [11]. The interaction of bivalent cations (e.g., Ca^2^⁺, Ba^2^⁺, or Sr^2^⁺) with ALG hydrogels has been extensively studied [12]. However, the impact of various anions on the characteristics of alginate hydrogels and the potential to create functional hydrogels with diverse properties by exploiting anion-specific effects have not been thoroughly investigated.

The calcium β-hydroxy-β-methylbutyrate (CaHMB), whose molecular formula is Ca(C_5_H_9_O_3_)_2_, is the most commonly supplemented and formulated form of β-hydroxy-β-methylbutyrate (HMB), a leucine-derived metabolite that maintains and improves muscle health [13]. In our previous study, we observed that the gel strength of ALG/CaHMB was greater than that of ALG/CaCl_2_ when both were at the same molar concentration of Ca^2+^ [14]. In order to better understand the crosslinking mechanism between CaHMB and sodium alginate, comparison with other calcium salts is necessary. Anions were selected as the ionic species because of their greater polarizability range, which leads to stronger ion-specific effects than the use of cations [15]. Anions have a stronger Hofmeister effect and are usually ordered as follows: CO_3_^2−^ > SO_4_^2−^ >S_2_O_3_^2−^ > H_2_PO_4_^−^ > F^−^ > CH_3_COO^−^ > Cl^−^ > Br^−^ > NO_3_^−^ > I^−^ >ClO_4_^−^ > SCN^−^ [8]. The series includes two different groups: SO_4_^2−^ ions are considered hydrated and polarizable (kosmotropic) anions, while I^−^ ions are considered dehydrated and highly polarizable (chaotropic) anions in most Hofmeister series classifications [16]. Consequently, the incorporation of both inorganic and organic calcium salts [17], together with various physical crosslinking methods, has led to the ongoing development of a wide range of ALG hydrogel formulations with distinct properties, markedly widening their scope of applications. However, the specific ionic effects of C_5_H_9_O_3_^−^ are unknown.

In this study, diverse monovalent anions (SO_4_^2−^, C_5_H_9_O_3_^−^, I^−^, and Ca^2+^) were added as constant counterions to the anionic polysaccharide alginate at three M/G ratios, and the anion-specific effects on the hydrogels were systematically analyzed in terms of the gelation time, gelation process, chemical structure, microstructure, moisture, and thermodynamic characteristics. The effects of different calcium salts and M/G ratios on the gelation behavior and properties of the alginate hydrogels were investigated. The results demonstrated that the choice of calcium source is crucial for the preparation of alginate hydrogels and can impact gelation and thermodynamic properties. This study highlights the importance of anion-specific effects on the characteristics of ALG hydrogels and provides insights into the utilization of these effects to fabricate functional hydrogels with variable properties.

## 2. Materials and Methods

### 2.1. Materials

The three alginates were procured from Macklin Biochemical Co., Ltd. (Shanghai, China). The structural differences among the three alginates are listed in Table 1. The alginate samples were designated as A21, A11, and A12, where the numerical postfix represents the M/G ratio of ALG. Calcium sulfate [CaSO_4_, purity ≥ 99.99%, molecular weight (Mw) = 136.14], calcium iodide (CaI_2_, purity = 99.999%, Mw = 293.89), and CaHMB (purity > 97%, Mw = 274.33) were obtained from Macklin Biochemical Co. Ltd. (Shanghai, China). All chemical reagents used in the experiments were of an analytical grade.

### 2.2. Preparation of Calcium Alginate Hydrogels

First, the ALG powder was dissolved in deionized water at 70 °C for 30 min until it was completely dissolved. Three calcium sources (CaSO_4_, CaI_2_, and CaHMB) were ultrasonicated at 25 °C for 30 min to allow for complete dissolution and storage at room temperature. The ALG solution and calcium source solution were then mixed equally so that the ALG and calcium ion concentrations in the final system were 5 mg/mL and 7.29 mM, respectively. Finally, the hydrogels were maintained at 25 °C for subsequent analysis.

### 2.3. Gelation Time

Sol–gel biotransformation was carried out after mixing prefilled ALG with CaSO_4_, CaI_2_, or CaHMB. To obtain a homogeneous mixture, the mixture was shaken for 30 s. A vial inversion test was used to measure the gelation time. The gelation rate was determined by the gelation time, which is the point at which the mixture ceases to flow when the *vial* is tilted.

### 2.4. Rheology Tests

The rheology was characterized by Wang et al., 2024, for hydrogels with modifications using a DHR-1 rheometer (TA Instruments Menu Co., Ltd., New Castle, DE, USA) [14]. After rapid mixing with the three calcium source solutions, the ALG/calcium salts solutions were quickly transferred to a rheometer, preset at 25 °C to monitor the gelation process. The storage modulus (*G*′) and loss modulus (*G*″) were recorded for 0–3600 s using the time-sweep dynamic oscillation mode at 0.5% stress and a frequency of 1 Hz. Calcium alginate hydrogels were spread between the spaces of the plate after 8 h at 0.1–10 Hz.

### 2.5. FTIR

The FTIR spectra of the freeze-dried powder of the hydrogels were recorded in the range of 4000–450 cm^−1^ using a Fourier transform infrared spectrometer (PerkinElmer Co., Ltd., Tokyo, Japan). All spectra were collected with a resolution of 4 cm^−1^ using 32 scans.

### 2.6. Cryo-SEM

The hydrogels were placed on a copper sample rack with a slight upward protrusion to facilitate freezing and breakage. The hydrogels were crushed and then placed in a vacuum environment in a PP3010T cryo-SEM preparation system (Quorum Technologies, Laughton, UK). The sublimation temperature was set to −90 °C for 30 min to facilitate the sublimation of water and prevent the cross-section from being encapsulated by ice [18]. The samples were observed and photographed using an SU8000 scanning electron microscope (Hitachi Co., Ltd., Tokyo, Japan) at 10.0 kV after sputtering with platinum at 5 mA for 120 s.

### 2.7. LF-NMR Relaxometry

LF-NMR relaxometry measurements were performed using a MesoQMR23-060H instrument (Niumag Electric Corporation, Shanghai, China). Approximately 2 g of the calcium alginate hydrogels was placed in an NMR tube (60 mm diameter) and inserted into the analyzer. The pertinent parameters were TW = 3000 ms, *τ* = 100, and NECH = 18,000, and the total number of echoes was 10,000. The *T_2_* values of the hydrogels were exponentially fitted, and measurements were acquired using the Carr-Purcell-Meiboom-Gill (CPMG) sequence with MultiExp Inv analysis software (Version 1.06, Niumag Electric Corporation, Shanghai, China).

### 2.8. Thermodynamic Analysis

Thermal gravimetric analysis (TGA) was performed using a NETZSCH TG 209 F1 (Netzsch-Gerätebau GmbH, Selb, Germany) from 30 to 200 °C with a uniform ramp-up rate of 10 °C/min in a nitrogen atmosphere.

### 2.9. Statistical Analysis

The data are provided in the form of mean values ± standard deviations. SPSS 26.0 (IBM SPSS Statistics 26.0) was used to analyze significant differences between the means. For all the statistical analyses, the significance threshold was set at *p* < 0.05.

## 3. Results and Discussion

### 3.1. Effects of M/G Ratios and Calcium Salt Types on Gelation Time

**Figure 1:**
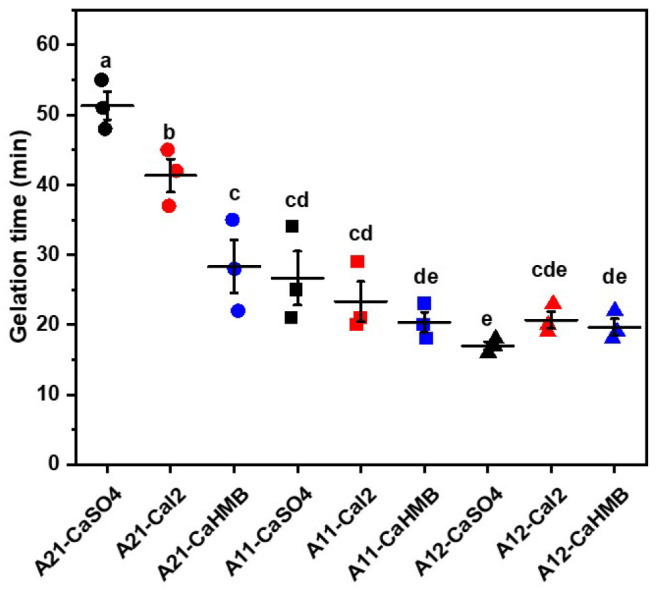
The gelation time of alginate hydrogels with different M/G ratios and calcium salts. The alginate samples were designated as A21 (●), A11 (■), and A12 (▲), where the numerical postfix represents the M/G ratio of alginate. Different lowercase letters (a, b, c, d, and e) indicate significant differences in gelation time (*p* < 0.05).

The gelation time of the calcium alginate hydrogels is shown in Figure 1 as a function of calcium salts (CaSO_4_, CaI_2_, and CaHMB) of ALG and the three M/G ratios, respectively. Improvements in the gelation time of the hydrogels were dependent on the type of alginate employed. The gelation time of A21-CaSO_4_ is 3.4 times longer than that of A12-CaSO_4_. Similarly, when M/G ratio was reduced from 2:1 to 1:1 for CaI_2_ and CaHMB, the gelation time significantly decreased. The M segment demonstrates a degree of flexibility and significant steric hindrance [3], which provides an obstruction to the crosslinking of Ca^2+^ and ALG (M/G = 2:1) for the prolongation of gel time. The G residues adopt an alternative ^1^C_4_ conformation, forming paired double structures that create a buckled cavity capable of trapping Ca^2+^ through a chelating binding mechanism. This cavity is shaped like an “egg-box” [12]. Because of the increase in the number of binding sites as the number of G residues increases, it is possible to hypothesize that random interchain crosslinking by Ca^2+^ cations occurs for the same amount of calcium released. The alginate gelation time was later, with the lowest amount of G residue. The effect of calcium source type was more significant (*p* < 0.05) in alginate hydrogels with M/G ratios of 2:1, in which the order of gelation time was A21-CaSO_4_ > A21-CaI_2_ > A21-CaHMB. Similar results were observed for the effects of calcium carbonate and calcium acetate on alginate-based pellets. The incorporation of calcium salts into pellet formations depends on the solubility of the calcium salts [19]. The incorporation of calcium salts (CaSO_4_, CaI_2_, and CaHMB) is related to the importance of calcium ions in the alginate gelling mechanism. The variation in gelation time observed among the different calcium salts can be attributed to their dissolution behavior in water. The gelation and physical properties of the hydrogels were affected by the addition of calcium salts (as discussed later).

### 3.2. Effects of the M/G Ratios and Calcium Salt Types on the Gelation Process

**Figure 2:**
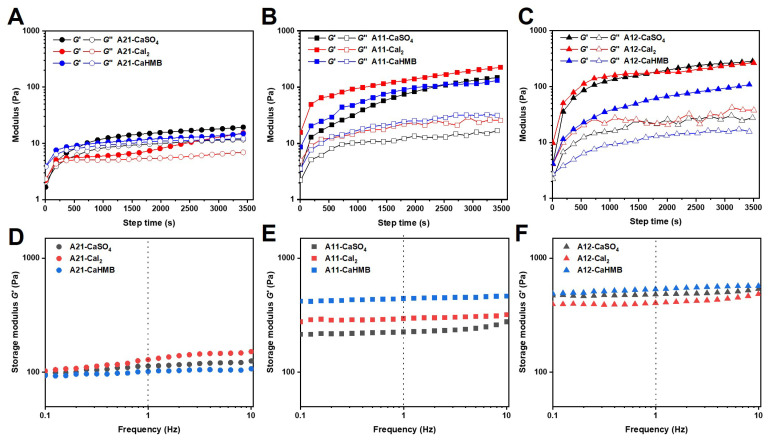
A time sweep of alginate hydrogels with different calcium salts at M/G ratios of (**A**) 2:1, (**B**) 1:1, and (**C**) 1:2. Storage modulus as a function of frequency for alginate hydrogels with different calcium sources at M/G ratios of (**D**) 2:1, (**E**) 1:1, and (**F**) 1:2. The alginate samples were designated as A21, A11, and A12, where the numerical postfix represents the M/G ratios of the alginate.

The rheological characterization of gelation mechanisms is of crucial importance for understanding the correlation between the microstructure and product properties. The gelation process, based on calcium salts (CaSO_4_, CaI_2_, and CaHMB) of ALG and the three M/G ratios of ALG, is shown in Figure 2A–C. The gelation process was classified into three stages: the initial period of gelation (*G*′ and *G″* increased exponentially with time and *G″* > *G*′), the gelation period (*G*′ increased faster than *G″* with exponentially increasing time and *G″* < *G*′) and the post-gel period (*G*′ and *G″* increased slowly with time).

The gel point (t*), which indicates the transition from a viscous liquid to a solid state, is one of the key kinetic characteristics of any crosslinking reaction. By assessing the intersection of *G’* and *G”*, t* can be obtained using a time sweep [20]. In these calcium alginate hydrogels, a solid phase clearly formed, and the crossover point suggested that the sample underwent “solidification” and became progressively more elastic. This can be explained by the increase in the number of calcium ions that are chelated and the appearance of a solid phase in the total mass. Moreover, the t* values of A21-CaSO_4_, A21-CaI_2_, and A21-CaHMB were 73.53 ± 10.84, 44.46 ± 9.68, and 6.50 ± 0.54 s, respectively. According to the Hofmeister series, anions and cations in solution form solvated shells via hydrogen bonding with water molecules. In general, when precursor salts dissociate into anions and cations, anions have a greater effect on accelerating gelation than cations [21]. The strength of the hydration interaction with water molecules was SO_4_^2−^ > I^−^. The C_5_H_9_O_3_^−^ comprises hydroxyl and carboxyl functional groups, which are capable of hydrogen interactions with water molecules or polysaccharide chains. However, the microstructure develops faster owing to the availability of more Ca^2+^ ions in CaHMB for crosslinking and diffusional limitations because of the formation of the microstructure. Therefore, a shorter period is required before t*. The gelation of ALG (M/G = 2:1) was affected by the variation in the calcium sources. These results were in good agreement with the gelation time measured using the *vial* inversion method (Figure 1).

In many gelation systems, it has been observed that gelation kinetics slow with time. The physical binding of Ca^2+^ ions with available G-blocks on neighboring alginate chains might explain the initial sharp increase in *G*′. Although *G′* continued to increase after t*, the rate of increase slowed, as seen from the change in the slope. Diffusion limitations caused by the growing “egg-box” microstructure are responsible for the change in kinetics [22]. The changes in the *G’* of the calcium alginate hydrogel after 8 h, as a function of frequency with calcium salts (CaSO_4_, CaI_2_, and CaHMB) of ALG and the three M/G ratios, are shown in Figure 2D–F. A crosslinking time of 8 h had a more significant impact on rheological properties compared to a shorter crosslinking time of 1 h (Figure 2). The *G*′ of A21-CaSO_4_, A21-CaI_2_, and A21-CaHMB at 1 Hz increased 5.84, 8.40, and 8.33 times, respectively, with increasing crosslinking time. When free Ca^2+^ ions combine with available G-blocks from neighboring chains to form crosslinks, the mobility of the alginate chains is significantly reduced. Calcium ions must diffuse through the developing structure to bind to vacant G-bonds and promote structural growth. As the complexity of the crosslinked network increases, the diffusion of free Ca^2+^ ions is hindered, resulting in a decrease in the growth rate of *G*′ [22]. Therefore, the hydrogel develops more slowly because of the limited availability of Ca^2+^ ions for crosslinking caused by the reduced amount of CaSO_4_ and diffusion limitations due to the microstructure’s formation.

Once all available G-blocks had bound Ca^2+^ ions, the plateau modulus of the system was reached. Therefore, it is likely that the plateau modulus is a function of the hydrogel composition. Hydrogels are 3D networks formed from polymer molecular chains through ionic interactions, *van der Waals* forces, hydrogen bonding, and hydrophobic interactions [21]. The final *G*′ values were in the order of A21-CaI_2_ > A21-CaSO_4_> A21-CaHMB. This result can also be considered a salt-out order in the Hofmeister series. The strength of the hydrophobic interaction is, therefore, dependent on the type of ions present, as the addition of salt promotes hydrophobic interactions through the salt-out effect [21]. The strength of the gel is closely related to the strength of the hydrophobic interactions. At the same concentrations of added calcium salt, A11-CaHMB had the highest final *G*′, followed by CaI_2_ and then CaSO_4_. A decreased degree of crosslinking may have influenced the decrease in *G*′ values. The released Ca^2+^ was insufficient for crosslinking because of the incomplete dissolution of calcium sources [23]. When the content of G residue continued to increase, there were enough carboxyl groups in the system to bind with calcium sources, resulting in the frequency curves of A12-CaHMB and A12-CaSO_4_ being as close as possible to dammit, indicating that the influence of calcium source dissolution on crosslinking decreased. The *G*′ value is a parameter that indicates the strength of the hydrogel [24]. For calcium ALG-based hydrogels formed using the same kind of calcium source, the higher the content of G residue in ALG, the greater the hydrogel strength.

### 3.3. Effects of M/G Ratios and Calcium Salt Types on Chemical Structures

**Figure 3:**
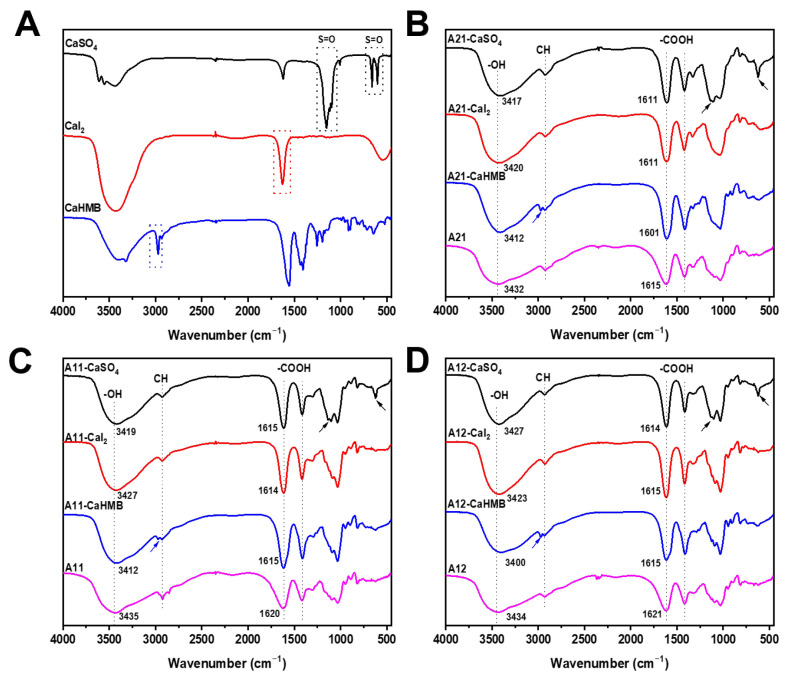
FTIR spectra of alginate hydrogels with different calcium sources at M/G ratios of (**A**) different calcium salts (**B**) 2:1, (**C**) 1:1, and (**D**) 1:2. The alginate samples were designated as A21, A11, and A12, where the numerical postfix represents the M/G ratio of alginate. The blue arrows indicate the characteristic peak positions of CaHMB.

The FTIR spectra are shown in Figure 3 for the calcium alginate hydrogel as a function of the M/G ratios (2:1, 1:1, 1:2) of ALG and the type of calcium source (CaSO_4_, CaI_2_, or CaHMB). The FTIR spectra of the three calcium sources are shown in Figure 3A. In the spectra of pristine ALG with different M/G ratios, the bands at 2923–2928 cm^−1^ and 3432–3435 cm^−1^ represent the stretching vibrations of C-H and O-H, respectively [25]. The band attributed to unionized carboxyl groups was approximately 1620 cm^−1^, whereas the corresponding band attributed to ionized carboxyl groups was approximately 1417 cm^−1^ [26]. Additionally, bands at approximately 800 and 700 cm^−1^ could be assigned to M and G acids, respectively, both of which are present in the ALG structure. Furthermore, the C-H stretching vibration of ALG/CaHMB exhibited a shift from a single peak to two distinct peaks. This may be attributed to the distinctive peak of CaHMB, which manifested at an approximate wavelength of 2970 cm^−1^ (Figure 3A). The strong peak at 1152 cm^−1^ and multiple peaks at 660 cm^−1^ and 601 cm^−1^ were attributed to the antisymmetric stretching and bending vibrations of S=O in SO_4_^2−^ [27]. These absorption bands in ALG-CaSO_4_ with different M/G ratios shifted to 1138 cm^−1^ and 622 cm^−1^, respectively. However, the characteristic peak of CaI_2_ was observed at 1627 cm^−1^, which shifted to approximately 1614 cm^−1^ in the ALG-CaI_2_ gel system.

The O-H stretching vibration peaks of the three calcium alginate hydrogels shifted to lower wavelengths. The O-H group characteristic peak redshifted owing to the establishment of -O→Ca^2+^ coordination bonds and the decreased number of hydrogen bonds between hydroxyl functional groups [28]. Moreover, the absorption peak in the 3200–3500 cm^−1^ region tended to shift to different extents immediately after the addition of calcium salts. CaHMB exhibited a maximum difference in wavenumber, followed by CaSO_4_ and CaI_2_, suggesting that more hydrogen bonds might be generated within the hydrogels by the addition of CaHMB [29]. In addition, the loose interactions (with lower cooperativity) are based on a hydrogen bond network involving MM blocks [30]. The frequencies of the *ν_asym_*(COO^−^) and *ν_sym_*(COO^−^) bands in the FTIR spectrum (1350–1750 cm ^−1^) are highly sensitive to the structure of the carboxylate group, the nature of the solvent, the nature of the ligand, and the identity of the metal ion; therefore, the separation of the bands [i.e., Δ*ν* = *ν_asym_*(COO^−^) and *ν_sym_*(COO^−^)] is also indicative of the structure of a given carboxylate [31]. The values of Δ*ν* for all the hydrogels were almost equal to or less than those of ALG (Table 2). The values of Δ*ν_hydrogel_*, like those of Δ*ν_ALG_*, can be correlated with bidentate bridging coordination. The carboxylic oxygen that is coordinated with the metal is hydrogen bonded to other ligands [31]. In contrast, the difference in Δ*ν* in CaHMB is increased, indicating an enhanced symmetry between the two oxygen atoms in the carboxyl group of the bidentate or bridged coordination caused by the hydrogen bond between HMB and the polysaccharide chain. Therefore, these results suggest that the shorter t* of A21-CaHMB might be related to stable chain–chain interactions in many hydrogen bond networks, including -COOH and -OH groups, serving as the basis for cross-bonding.

### 3.4. Effects of the M/G Ratios and Calcium Salt Types on Microstructure

**Figure 4:**
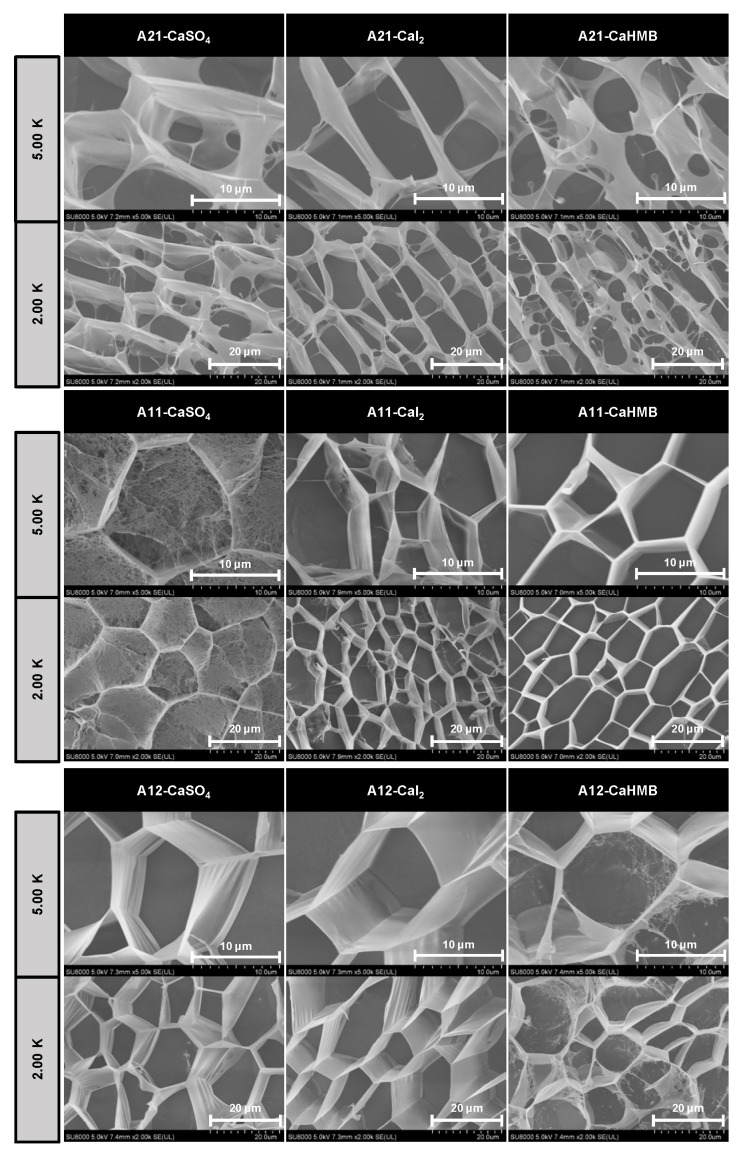
The micromorphology of alginate hydrogels with different calcium sources at varying M/G ratios. The images were acquired at magnifications of 5.00 K (scale bar, 10 μm) and 2.00 K (scale bar, 20 μm). The alginate samples were designated as A21, A11, and A12, where the numerical postfix represents M/G ratio of the alginate.

Cryo-SEM provides the native conformation of the hydrogel because rapid freezing converts the sample interstitial water into crystal ice. Cryo-SEM images of the calcium alginate hydrogel, as a function of the M/G ratios (2:1, 1:1, 1:2) of ALG and the type of calcium source (CaSO_4_, CaI_2_, and CaHMB) at different magnifications (5.00 K and 2.00 K), revealed 3D network structures with honeycomb and stretching pores (Figure 4).

The hydrogels exhibited a mesh structure characteristic of typical hydrogel matrices. The presence of calcium salts appeared to affect the crosslinking of ALG. The porosity of the alginate is important for changing the alginate characteristics (M/G ratios) and gelling kinetics, and the resulting alginate gel may be manipulated to form a porous matrix upon gelation. Compared with the A11-CaI_2_ and A11-CaSO_4_ hydrogels, the A11-CaHMB hydrogels demonstrated greater mechanical strength owing to their more uniform and compact structures. The gelation behavior is mainly attributed to the interactions between Ca^2+^ and G residues, resulting in the crosslinking of polymer chains via the formation of junction zones [32]. In addition, the dependence of the ALG network density on the M/G ratio corresponded to the higher *G*’ value observed (Figure 2E); that is, gels with ALG (M/G = 1:2) were stronger than those with ALG (M/G = 2:1). The gel pore walls of A21-CaI_2_ are more complete. Adjusting molecular components not only controls mechanical properties, but also the microstructure of the gel. This, in turn, influences its macroscopic properties [33]. The presence of CaSO_4_ and CaHMB appeared to disrupt the continuity of the ALG (M/G = 2:1) network strands, and the matrix exhibited an increased degree of broken and/or interrupted junctions, as expected from the decreased modulus of the ALG (M/G = 2:1) systems. Observations were made on the differences in network density and walls within ALG (M/G = 2:1) for each calcium salt. This was related to the strength of the interaction between the calcium salt anion groups during ALG (M/G = 2:1) formation. The hydroxyl and carboxyl groups on the ALG (M/G = 2:1) chains form many hydrogen bonds. We discuss this mechanism using the Hofmeister series. The ions, including SO_4_^2−^ and C_5_H_9_O_3−_ (anion group of CaHMB), induce the salting-out phenomenon of the polymers. This resulted in the collapse of the polymer chains and the subsequent formation of small pores. The salting-in phenomenon caused by ions such as I^−^ leads to the formation of larger pores [6]. Compared to SO_4_^2−^ at the same salinity, weakly hydrated I^−^ has a chaotropic nature, which increases colloidal stability by increasing the hydration of ALG (M/G = 2:1) and creating a repulsive force. Therefore, the entry of SO_4_^2−^ and C_5_H_9_O_3_^−^ can polarize hydrated water molecules and interfere with the hydrophobic hydration of macromolecules by increasing the surface tension of the cavity surrounding the backbone.

### 3.5. Effects of the M/G Ratios and Calcium Salt Types on Moisture Migration

**Figure 5:**
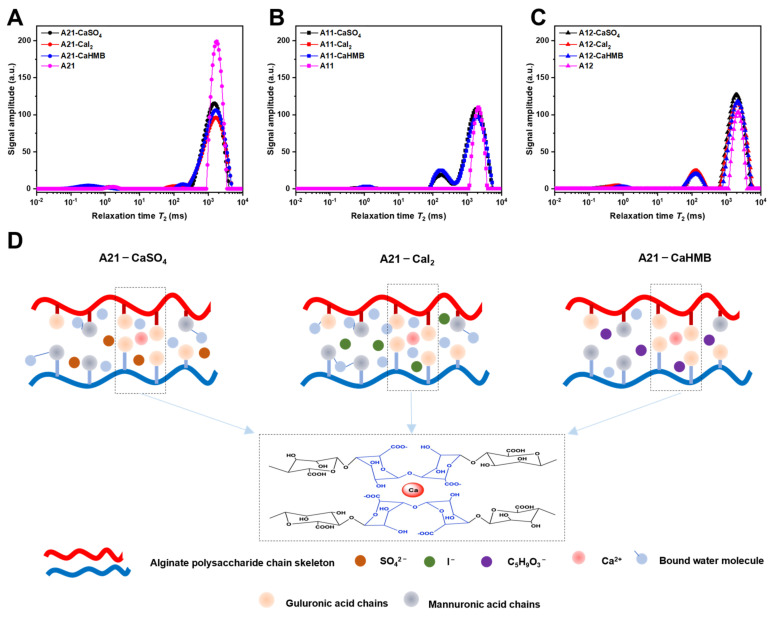
Moisture characteristics of alginate hydrogels with different calcium sources. *T_2_* spectra of alginate hydrogels with different calcium sources at M/G ratios of (**A**) 2:1, (**B**) 1:1, and (**C**) 1:2. (**D**) A schematic illustration of the removal of water from the polymer backbone in the presence of different ionic species. The alginate samples were designated as A21, A11, and A12, where the numerical postfix represents the M/G ratio of alginate.

The *T*_2_ spectra of the ALG samples with calcium salts (CaSO_4_, CaI_2_, and CaHMB) of ALG and the three M/G ratios are summarized in Figure 5 and Table 3. A short *T*_2_ is assigned to the hydrogen nuclei in an immobile structure, whereas a long *T*_2_ is assigned to the hydrogen nuclei in a mobile structure. One component (*T*_23_) was observed in the relaxation spectrum for the ALG (M/G = 1:1) and ALG (M/G = 1:2) solutions, whereas two components (*T*_21_ and *T*_23_) were observed for ALG (M/G = 2:1). The M residues of ALG are preferentially “mobilized” during the controlled hydration process [34]. The shorter relaxation time (*T*_21_) of ALG (M/G = 2:1) reflected the presence of water, which was closely related to the M residues on alginate.

The transverse relaxation times of the different water states in the calcium alginate hydrogel are represented by *T*_21_, *T*_22_, and *T*_23_. *T*_21_ and *T*_22_ have little effect on the strength and water-binding capacity of the gel, representing water that is closely associated with macromolecules and weakly bound water in the gel network, respectively. The *T*_23_ component is attributed to water, which can be easily extracted from the gel structure [35]. As shown in Figure 5A and Table 3, the percentage of free water was the highest, whereas the percentages of loosely bound water and tightly bound water were comparable. The shorter the *T*_2_, the less mobile the water fraction. This results in a faster rate of relaxation time. The inverse is also true. The addition of calcium ions reduces the mobility of the system to various types of water. The network structure facilitates the absorption of a substantial quantity of water, which influences the distribution and concentration of water within the hydrogel [36]. Compared with that of A21-CaSO_4_, the *T*_21_ of A21-CaI_2_ moved toward a lower relaxation time, and the mobility of the tightly bound water was lower. SO_4_^2−^ is a well-hydrated ion, and the increase in its surface tension suggests that the removal of water from the polymer backbone has a significant impact on the gelation process. I^−^ is a chaotropic or poorly hydrated ion [33]. CaHMB, as a calcium source, had the maximum mobility of tightly bound water, indicating that C_5_H_9_O_3_^−^ is a well-hydrated ion and its hydration capacity is greater than that of SO_4_^2−^. It was also found that, in the ALG (M/G = 2:1) hydrogels, the use of loosely reduced the relaxation time. This phenomenon can also be attributed to the diffusional exchange of free and tightly bound water at the polymer-binding site, which controls the presence of loosely bound water. Different calcium sources also had a significant effect on *T_21_*. During the crosslinking process, the carboxyl group of alginate binds to Ca^2+^ ions, resulting in an “egg-box” structure. This structure reduces the availability of alginate chains for binding to water molecules, thereby reducing the hydrophilicity of the material [37]. As a calcium source, CaHMB had the maximum mobility of tightly bound water, indicating that greater numbers of “egg-box” structures were produced and that the hydrophilicity was reduced. However, with the increase in G residue content, the free water and tightly bound water in the system were transformed into loosely bound water (Figure 5B,C). The hydration of calcium salt anion gradually weakened. When the M/G ratio was increased to 1:2, the hydrophilicity of the polysaccharide chain was lowered due to the increase in “egg-box” structures. Based on the analysis described above, it is suggested that the mechanism of the enhancement of the hydrophobicity of the calcium alginate hydrogels at the M/G ratio of 2:1 is different depending on the ionic species (Figure 5D). The greater the hydration of the anion in calcium salts such as CaHMB and CaSO_4_, the more water can be removed from the alginate polysaccharide backbone, resulting in the increased mobility of the tightly bound water.

### 3.6. Effects of M/G Ratios and Calcium Salt Types on Thermodynamic Analysis

**Figure 6:**
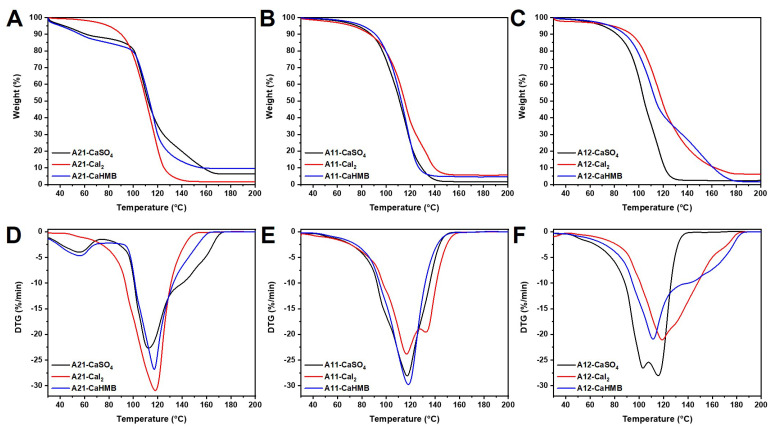
Thermodynamic analysis of alginate hydrogels with different calcium sources. Thermal gravimetric analysis of alginate hydrogels at M/G ratios of (**A**) 2:1, (**B**) 1:1, and (**C**) 1:2. Differential TG (DTG) of alginate hydrogels with M/G ratios of (**D**) 2:1, (**E**) 1:1, and (**F**) 1:2. Alginate samples were designated as A21, A11, and A12, where numerical postfix represents M/G ratio of alginate.

Thermo gravimetric analysis (TGA) is a thermodynamic technique that measures the relationship between the weight and temperature of hydrogels during controlled heating to assess their thermal stability [38,39]. Thermal gravimetric analysis (TGA) and differential TG (DTG) analyses were performed to investigate how processing affects the thermal decomposition of calcium alginate hydrogels with calcium salts (CaSO_4_, CaI_2_, and CaHMB) of ALG at three M/G ratios, and the curves of the samples are presented in Figure 6. The corresponding TGA and DTG curve data, such as the initial decomposition temperature at 5% weight loss (T_5%_) and 10% weight loss (T_10%_), the maximum rate of decomposition temperature (T_max_), and amount of char remaining at 200 °C, are shown in Table 4.

The pyrolysis process of ALG hydrogels was divided into two stages. The first stage was attributed to crystal water release and moisture evaporation from 30 to 100 °C [40]. Within this temperature range, approximately 9–16% weight loss was observed, demonstrating the hydrophilic nature of the hydrogels and the interactions between water molecules and the surface hydroxyl groups of ALG, CaSO_4_, CaI_2_, and CaHMB. As shown in Figure 6A, the decomposition stages of A21-CaI_2_ are slightly different. In contrast to the low weight loss observed for A21-CaI_2_ in the initial stage, A21-CaSO_4_ and A21-CaHMB exhibited higher weight loss rates, reaching 12 and 16%, respectively, during the initial thermal degradation stage at temperatures below 100 °C. In particular, the T_5%_ and T_10%_ significance of A21-CaHMB was lower than that of A21-CaI_2_ and A21-CaSO_4_ (*p* < 0.05, Table 4). The results of thermogravimetric analysis show that the water of A21-CaHMB is more easily decomposed by heating, which is consistent with the results of LF-NMR and microstructure analysis. Meanwhile, these results are consistent with those of LF-NMR, and the mobility of A21-CaI_2_ to tightly bind and loosely bind water was the lowest (Table 3). Because the COONa groups of ALG are bound by Ca^2+^ ions, the hydrophilicity of the polysaccharide chain decreases. For this reason, the ALG (M/G = 1:1) hydrogels and ALG (M/G = 1:2) hydrogels shown in Figure 6B-C lost less weight than the calcium alginate hydrogel (M/G = 2:1) in the first stage. The second stage of degradation occurs at temperatures higher than 100 °C, which is mainly due to the multiple stages of calcium alginate degradation [41]. Degradation occurred in two stages, the first of which took place between 100 and 120 °C, possibly by breaking down the alginate carbon chains to form an intermediate product. The weight loss increased rapidly during this stage. The final stage, associated with the decomposition of the middle phase, occurred at temperatures above 120 °C [42]. Therefore, different types of calcium salts had a greater influence on the thermal stability of calcium alginate hydrogels at higher M/G ratios. The higher the content of the M residue of ALG was, the stronger the hydration ability of the anion in the calcium sources (CaHMB > CaSO_4_ > CaI_2_) and the faster the thermal degradation of the water in the calcium alginate hydrogel.

## 4. Conclusions

The type of calcium salt (CaSO_4_, CaI_2_, and CaHMB) and the three M/G ratios affect the gelation, moisture, and thermodynamic properties of calcium alginate hydrogels. In particular, the ionic effect of the calcium salt anions was found to be more significant on calcium alginate hydrogels (M/G = 2:1). The increasing number of M fragments hindered the binding of Ca^2+^ and GG fragments, but CaHMB accelerated the binding and enhanced the gel strength. Strongly hydrated ions (such as SO_4_^2−^ and C_5_H_9_O_3_^−^) cause the removal of water molecules from polysaccharide chains, and weakly hydrated chaotropic anions (I^−^) promote the binding of alginate polysaccharide chains and water molecules. However, due to the hydration of ions, the binding water of CaHMB is weakened and the thermal stability is reduced. The present study demonstrated that different calcium sources and M/G ratios strongly influence the formation of calcium alginate hydrogels, providing insights into a potentially complex system that may be utilized in the food industry.

## Figures and Tables

**Table 1:** Structural characterization of alginate.

Sample	M/G Ratios	Mw (g/mol)	Polydispersity Mw/Mn
A21	2:1	667622	1.36
A11	1:1	366279	1.01
A12	1:2	450057	1.11

**Table 2:** Carboxylate symmetric and asymmetric vibrational frequencies.

Sample	*ν_asym_*(COO^−^)	*ν_sym_*(COO^−^)	Δ*ν*(COO^−^)
A21-CaSO_4_	1607	1419	188
A21-CaI_2_	1611	1423	188
A21-CaHMB	1611	1416	195
A21	1615	1417	198
A11-CaSO_4_	1615	1416	199
A11-CaI_2_	1614	1417	197
A11-CaHMB	1615	1412	203
A11	1620	1417	203
A12-CaSO_4_	1614	1416	198
A12-CaI_2_	1615	1416	199
A12-CaHMB	1615	1412	203
A12	1621	1417	204

**Table 3:** Relaxation time of calcium alginate hydrogel.

Sample	*T*_21_	*T*_22_	*T*_23_
A21-CaSO_4_	1.55 ± 0.06 ^a^	170.37 ± 6.91 ^ab^	1465.76 ± 59.41 ^a^
A21-CaI_2_	1.33 ± 0.19 ^b^	158.94 ± 6.44 ^b^	1607.91 ± 63.69 ^b^
A21-CaHMB	2.05 ± 0.17 ^c^	174.36 ± 6.91 ^a^	1607.91 ± 63.69 ^b^
A21	1.74 ± 0.07 ^d^	-	1762.91 ± 0.00 ^c^
A11-CaSO_4_	1.13 ± 0.16 ^a^	174.36 ± 6.91 ^a^	1847.41 ± 73.17 ^a^
A11-CaI_2_	1.03 ± 0.15 ^a^	162.66 ± 6.44 ^ab^	1980.22 ± 78.43 ^b^
A11-CaHMB	1.14 ± 0.20 ^a^	158.94 ± 6.44 ^b^	2025.50 ± 0.00 ^bc^
A11	-	-	2122.58 ± 84.07 ^c^
A12-CaSO_4_	0.53 ± 0.06 ^a^	138.34 ± 5.61 ^a^	1847.41 ± 73.17 ^a^
A12-CaI_2_	0.71 ± 0.08 ^b^	138.79 ± 14.94 ^ab^	2122.58 ± 84.07 ^b^
A12-CaHMB	0.93 ± 0.04 ^c^	123.22 ± 4.88 ^b^	2074.04 ± 84.07 ^b^
A12	-	-	2122.58 ± 84.07 ^b^

The alginate samples were designated as A21, A11, and A12, where the numerical postfix represents the M/G ratios of the alginate. The data are expressed as the means ± SDs from triplicate determinations. The presence of different letters (^a^, ^b^, ^c^, and ^d^) in the same column indicates significant differences with different M/G ratios (*p* < 0.05).

**Table 4:** Thermal degradation data of calcium alginate hydrogel in nitrogen.

Sample	T_5%_ (°C)	T_10%_ (°C)	T_max_ (°C)	Residue (%) at 200 °C
A21-CaSO_4_	46.0 ± 2.5 ^a^	61.3 ± 0.4 ^a^	112.0 ± 2.0 ^a^	4.3 ± 1.1 ^a^
A21-CaI_2_	80.8 ± 1.5 ^b^	89.7 ± 0.5 ^b^	117.5 ± 1.4 ^b^	3.3 ± 1.9 ^a^
A21-CaHMB	39.8 ± 0.5 ^c^	55.2 ± 1.5 ^c^	117.0 ± 0.8 ^b^	6.3 ± 2.0 ^a^
A11-CaSO_4_	76.7 ± 0.2 ^a^	88.3 ± 0.7 ^a^	117.3 ± 0.7 ^a^	3.0 ± 1.4 ^a^
A11-CaI_2_	73.9 ± 2.5 ^a^	87.7 ± 1.0 ^a^	116.4 ± 1.4 ^a^	4.6 ± 1.0 ^a^
A11-CaHMB	79.9 ± 1.6 ^a^	90.7 ± 1.3 ^a^	118.0 ± 1.1 ^a^	4.5 ± 1.3 ^a^
A12-CaSO_4_	72.6 ± 1.8 ^a^	83.4 ± 1.3 ^a^	115.1 ± 1.8 ^a^	3.7 ± 1.4 ^a^
A12-CaI_2_	78.5 ± 2.2 ^a^	94.2 ± 1.6 ^b^	117.0 ± 1.5 ^a^	4.3 ± 1.0 ^a^
A12-CaHMB	76.1 ± 1.2 ^a^	88.7 ± 1.0 ^c^	113.4 ± 1.9 ^a^	2.8 ± 1.3 ^a^

The alginate samples were designated as A21, A11, and A12, where the numerical postfix represents the M/G ratios of the alginate. The data are expressed as the means ± SDs from triplicate determinations. Different letters (^a^, ^b^, and ^c^) in the same column indicate significant differences with different M/G ratios (*p* < 0.05).

## Data Availability

The original contributions presented in the study are included in the article, further inquiries can be directed to the corresponding author.

## Abbreviations

ALG	alginate
CaHMB	calcium β-hydroxy-β-methylbutyrate
M/G ratios	mannuronic/guluronic acids ratios
LVR	linear viscoelastic region
FTIR	Fourier transform infrared spectroscopy
cryo-SEM	cryo-scanning electron microscopy
LF-NMR	low-field NMR
CPMG	Carr-Purcell-Meiboom-Gill sequence
*T*_2_	transverse spin-spin
TGA	thermal gravimetric analysis
DTG	derivative thermogravimetric
